# Coding-Complete RNA Virus Genomes Assembled from Murine Cecal Metatranscriptomes

**DOI:** 10.1128/MRA.00018-20

**Published:** 2020-03-19

**Authors:** Joshua M. A. Stough, Andrew J. Beaudoin, Patrick D. Schloss

**Affiliations:** a Department of Microbiology and Immunology, University of Michigan, Ann Arbor, Michigan, USA; DOE Joint Genome Institute

## Abstract

Efforts to catalog viral diversity in the gut microbiome have largely focused on DNA viruses, while RNA viruses remain understudied. To address this, we screened assemblies of previously published mouse gut metatranscriptomes for the presence of RNA viruses. We identified the coding-complete genomes of an astrovirus and five mitovirus-like viruses.

## ANNOUNCEMENT

The viral fraction of the mammalian gut microbiome forms a crucial component in the relationship between microbes and their host. Bacterial viruses serve as an important source of genetic diversity and population control for the microbiota, driving its ecology and evolution (1). Mammalian viruses disrupt the gut environment through infection and the response of the host immune system (2). Bacterial and mammalian viruses make significant contributions to host health and disease. Current efforts to describe the diversity of viruses present in the gut have focused on using shotgun metagenomics to identify double-stranded DNA viruses, predominantly bacteriophages and host pathogens (3). However, this method ignores viruses with RNA genomes, which make up a considerable portion of environmental viromes (4).

We reanalyzed deeply sequenced metatranscriptome data produced by our laboratory for the study of microbiome dynamics in a mouse model of Clostridioides difficile infection (5, 6). Briefly, C57BL/6 mice from a breeding colony that we maintain at the University of Michigan were treated with one of three different antibiotics (clindamycin, streptomycin, or cefoperazone). After a 24-h recovery period, the mice were infected with C. difficile strain 630. Germfree C57BL/6 mice were also monoassociated with C. difficile strain 630. Cecal contents were removed from each animal 18 h postinfection and frozen for RNA extraction and sequencing. RNA sequences from each sample were trimmed of adapter sequences and low-quality bases using Trimmomatic v0.39, assembled individually using rnaSPAdes v3.13.1 (7), and concatenated for dereplication, which resulted in 70,779 contigs longer than 1 kb. Contigs were screened for the presence of RNA-dependent RNA polymerase (RdRP) coding sequences using BLAST v2.9.0 against a database containing all viral RefSeq protein sequences annotated as RdRP (screening database available online, as described below), with a maximum E value of 10^−20^, which resulted in 29 contigs. RdRP is conserved among almost all RNA viruses without a DNA stage in genome replication. These contigs were then annotated with InterProScan v5.39-77.0 (8, 9). We constructed phylogenetic trees from RdRP protein sequences using IQ-TREE v1.6.12 (10).

**FIG 1:**
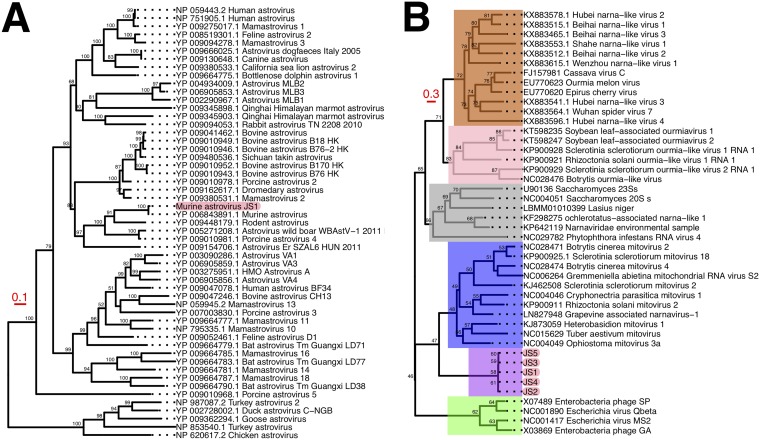
Phylogenetic trees showing the relatives of the metatranscriptome-assembled genomes. Maximum likelihood phylogenetic trees constructed from RdRP amino acid sequences for astroviruses (A) and narnaviruses (B) are shown. Node annotations represent IQ-TREE ultrafast bootstrap statistics; values less than 50% were excluded from the tree. Scale bars are indicated in red to the left of each tree. Highlight colors in panel B represent major *Narnavirus* taxa (orange, ourmiaviruses; pink, ourmia-like mycoviruses; gray, narnaviruses; blue, mitoviruses; purple, murine mitovirus-like viruses; green, leviviruses).

Two classes of RNA viruses were assembled, with high coverage, with sequences originating from most of the mouse treatment groups, including germfree mice. First, a 6,811-base-long astrovirus genome (GC content, 56.6%) was obtained with 1,683.5-fold coverage (Fig. 1A). The genome contained three predicted open reading frames, encoding a capsid, RdRP, and a trypsin-like peptidase, and appeared to be closely related to murine astroviruses in *Astroviridae*. Second, five distinct but closely related RNA virus genomes (designated putative mitovirus JS1 through JS5), ranging in length from 2,309 to 2,447 bases, with 4.6- to 16,078.8-fold coverage and an average GC content of 46.2%, belonged to a previously undescribed clade of *Narnaviridae* adjacent to the mitoviruses (Fig. 1B). These RNA virus genomes will facilitate future studies of RNA virus biology in the murine microbiome.

### Data availability.

The transcriptome sequencing (RNA-seq) data are available in the NCBI Sequence Read Archive (SRA) database under accession numbers PRJNA354635 (C. difficile-infected mice) and PRJNA415307 (mock-infected mice). The assembled genomes are available in GenBank under accession numbers MN780842 to MN780847. All of the scripts and software used to perform this analysis are available online (https://github.com/SchlossLab/Stough_Mouse_RNA_Virome_MRA_2019).
